# B‐lymphoblastic leukemia/lymphoma with DUX4 rearrangement

**DOI:** 10.1002/jha2.919

**Published:** 2024-05-09

**Authors:** Wing Kit Lam, Ching Ching Alice Wong

**Affiliations:** ^1^ Department of Clinical Pathology Tuen Mun Hospital Hong Kong Hong Kong

**Keywords:** acute leukemia, morphological, leukemia

1

A 35‐year‐old man presented with dizziness, exertional dyspnea, and palpitations for 1 month without fever. Complete blood count showed anemia (hemoglobin, 40 g/L), leucopenia (3.70 × 10^9^/L), and neutropenia (1.60 × 10^9^/L) with occasional circulating blasts on the peripheral blood smear. Bone marrow aspirate (May‐Grünwald‐Giemsa stain, ×1000; Figure 1, upper panels) showed 91% medium‐sized blasts with around half of them showing “cup‐like” nuclei (black arrowheads) and around a third of them showing cytoplasmic and/or nuclear blebs (black arrows). Some of the blasts showed both features (white arrows). Some leukemic cytoplasmic fragments were noted in the background (white arrowheads). Flow cytometry showed B‐lymphoblasts which demonstrate co‐expression of CD2 and CD371 (Figure 1, lower panel). Karyotype was normal. Next‐generation sequencing showed IKZF1 partial deletion (exons 4–7), PTPN11, and multiple NRAS mutations. Targeted RNA sequencing showed the presence of IGH::DUX4 fusion, confirming the diagnosis. The patient was given pediatric‐inspired intensive chemotherapy and achieved complete remission. He was planned to have allogeneic hematopoietic stem cell transplantation.

**FIGURE 1:**
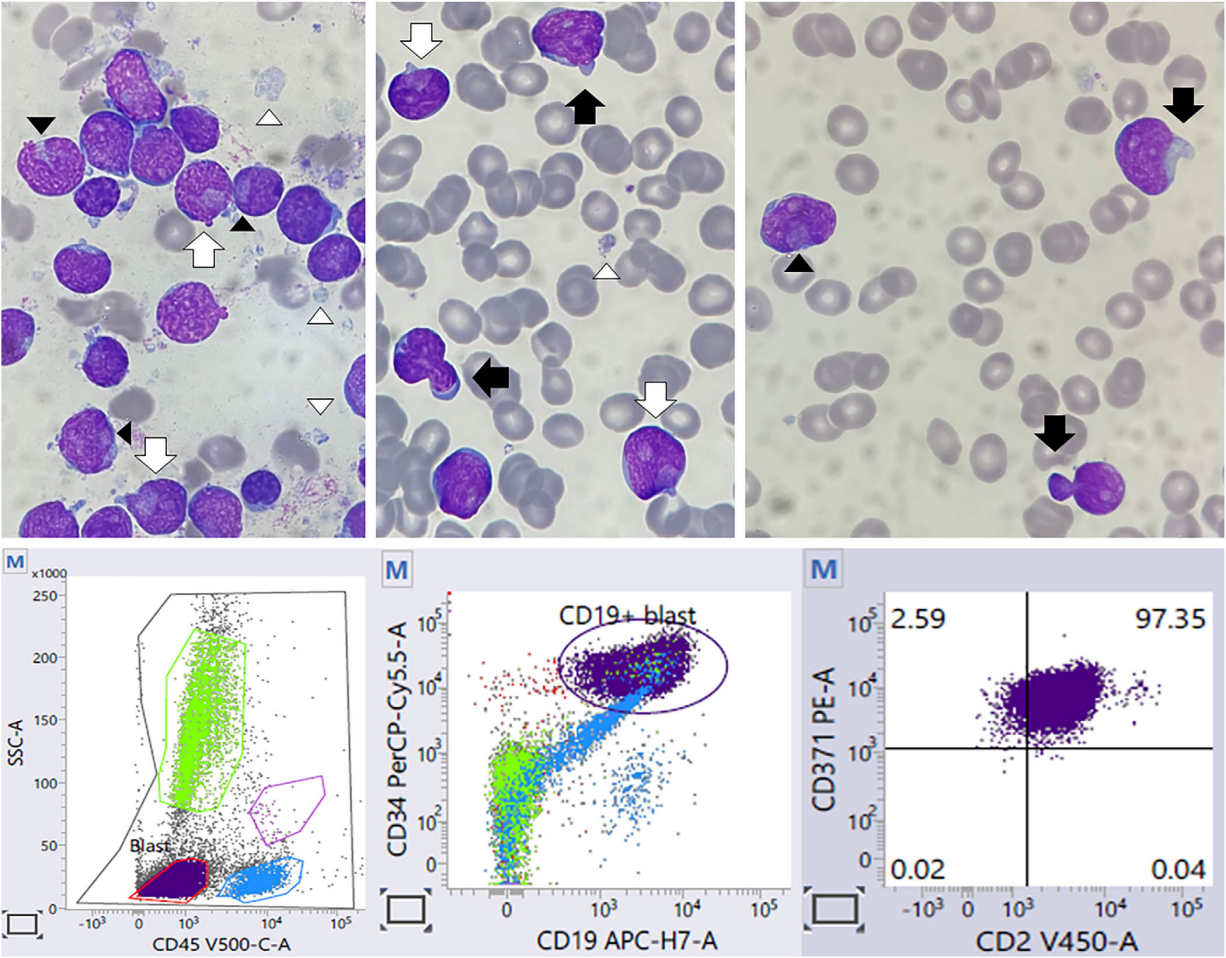
The morphological findings (upper panels) and the flow cytometry findings (lower panel) of the bone marrow aspirate of the patient.

B‐lymphoblastic leukemia/lymphoma (B‐ALL) with DUX4 rearrangement is a new provisional entity in the 5th edition of the World Health Organization Classification of Hematolymphoid Tumors which is more common in children, adolescents, and young adults and is associated with good prognosis. DUX4 rearrangements in B‐ALL are usually cytogenetically cryptic. Co‐expression of CD2 and CD371 in B‐ALL is strongly associated with DUX4 rearrangement. Yet, morphological description of this entity is scarce. “Cup‐like” nuclei in blasts are known to be associated with acute myeloid leukemia with NPM1 and/or FLT3‐ITD mutations but are less recognized in B‐ALL. Moreover, cytoplasmic and nuclear blebs are hitherto not described as distinctive features in any specific subtype of B‐ALL. Further study on the link between the morphological and molecular features of B‐ALL with DUX4 rearrangement cases would be of value.

## AUTHOR CONTRIBUTIONS

Wing Kit Lam analyzed the data, wrote the paper, and produced the figures. Ching Ching Alice Wong analyzed the data and wrote the paper.

## CONFLICT OF INTEREST STATEMENT

The authors declare no conflict of interest.

## FUNDING INFORMATION

No funding source was declared.

## ETHICS STATEMENT

The authors have confirmed ethical approval statement is not needed for this submission.

## PATIENT CONSENT STATEMENT

Written informed consent from the patient was obtained.

## CLINICAL TRIAL REGISTRATION

The authors have confirmed clinical trial registration is not needed for this submission.

## Data Availability

Data sharing is not applicable to this article as no new data were created.

